# Mental illness through the perspective of undergraduate medical students in Greece: a cross-sectional study at Aristotle University of Thessaloniki

**DOI:** 10.3389/fpsyt.2023.1228539

**Published:** 2023-10-31

**Authors:** Georgia-Nektaria Porfyri, Maria Athanasiadou, Vasileios Siokas, Konstantinos Angelopoulos, Sofia Skarpari, Sofia-Chrysovalantou Zagalioti, Efthimios Dardiotis, Jobst Rudolf, Georgia Deretzi, Anastasia Konsta, Ioannis Diakogiannis

**Affiliations:** ^1^First Psychiatric Clinic, “Papageorgiou” General Hospital, School of Medicine, Faculty of Health Sciences, Aristotle University of Thessaloniki, Thessaloniki, Greece; ^2^Department of Neurology, University Hospital of Larissa, Faculty of Medicine, School of Health Sciences, University of Thessaly, Larissa, Greece; ^3^Basic School of Medical Corps Hellenic Army, Athens, Greece; ^4^Department of Neurology, “Papageorgiou” General Hospital, Thessaloniki, Greece; ^5^Department of Emergency Medicine, AHEPA University General Hospital, Aristotle University of Thessaloniki, Thessaloniki, Greece

**Keywords:** stigma, mental health, mental illness, stigma reduction, students’ stigma, medical students’ stigma, Greek medical stigma, Greek students’ stigma

## Abstract

**Introduction:**

Numerous studies reveal that mental health-related stigma, stereotypes, and prejudices negatively affect the patients, jeopardizing their health, prognosis, and social opportunities. Healthcare professionals, who are in the first line of combating mental disease, are expected to play a significant role in drastically changing discriminatory and stigmatizing attitudes toward psychiatric patients and in diminishing the existing healthcare and social disparities. In this study, we aimed to explore and highlight the views of Greek medical students—that is of the future physicians—toward mental illness and people suffering from it.

**Materials and methods:**

It is a cross-sectional, observational study, in which 324 undergraduate students from the most populous Greek medical school of the Aristotle University of Thessaloniki, participated online, during the spring semester of 2022. The tools used were the Opinions about Mental Illness Scale (OMI) that assesses one’s viewpoints about mental illness, the Social Distance Scale (SDS) that captures the desired degree of social distancing from patients with mental disorders, and the Level of Contact Report (LCR-12) that estimates the level of familiarity with them.

**Results:**

Participants displayed rather positive attitudes regarding the etiology of mental illness, social integration, and discrimination toward psychiatric patients [as evaluated with the respective OMI subscales; Etiology mean score (μ):8.87 ± 4.68, Social Integration (μ):17.79 ± 5.42, Social Discrimination (μ):13.54 ± 11.17], and more clearly favorable opinions concerning the need for social provision or the enactment of restrictive measures [as expressed with the relative OMI subscales; Social Care (μ):22.74 ± 4.56, Social Restriction (μ):13.27 ± 8.98], while claiming to be quite familiar with mental disorders and individuals experiencing them (as assessed with LCR; μ: 8.71 ± 2.16), and relatively willing to interact with them (as measured with SDS; μ:8.95 ± 4.23). Degree of familiarity with mental illness was directly proportional to the desire for contact with patients living with it, while the higher both were, the more improved most of the aforementioned OMI sectors were found to be. Female sex, clinical medical education, previous clinical psychiatric training, and living with or being a person with a mental disorder were the factors that defined a statistically refined profile in many of the aspects above.

**Conclusion:**

Our findings are in accordance with many prior and recent studies, while showing improved opinions compared to those of previous research in Greek student and healthcare population. They are calling for vigilance, rather than complacency, as well as educational and social interventions, in order to enable current and future healthcare professionals to perform their function to its fullest extent. Implications of our results and further research suggestions are included.

## Introduction

Mental health-related stigma constitutes a global issue; there is no nation, community or culture where the psychiatric patients are treated as of equal societal worth to those considered mentally healthy (1). Historically, only rare health conditions like leprosy—with its alarming sight and contagiousness—had social effects comparable to those of mental health illnesses (2). Numerous studies reveal that attitudes toward psychiatric patients are often influenced by religion, ethnicity, and racial differences (3–6), by political characteristics and population density (7–10), as well as by culture, social norms, and values (11).

Accordingly, it should not be forgotten that individuals suffering from a mental disorder are forced to give an uneven battle not only against the condition itself but also against a “second disease”: the social stigma (12). This “social disease” is probably favored by the nature and intensity of acute psychiatric symptoms and is mainly based on the wide ignorance, the traditional superstitions (13) or even the misrepresentation of patients’ profile in the media and the arts and the subsequent fear created by these factors. The existence of stereotypical views—like the belief that psychiatric patients are unpredictable or menacing—contributes to the discrimination against them and the deprivation of their basic human rights, resulting in their repetitive exposure to major social disparities and isolation (14, 15).

In addition, the presence of stigmatizing perceptions concerning mental health disorders creates barriers for patients seeking care, due to their efforts to avoid the mentally ill’s label (16). As a result, stigma affects the self-esteem of psychiatric patients, prolongs their recovery, and burdens their physical health as well (17, 18), thereby jeopardizing their prognosis (19).

Healthcare professionals, who are on the frontline in the fight against mental illness ignorance and stigma, are expected to play a significant role in drastically changing discriminatory and stigmatizing attitudes toward people suffering from mental diseases (20). This includes advocating for these patients, by helping them on their anti-stigma efforts and campaigns, by co-educating with them the public that consider their opinion as expert (21, 22), by pressuring governments and organizations, and—last but not least—by supporting them actively in terms of accessibility and care of their mental and physical health. However, for them to successfully play this role, they should have received an anthropocentric, patient-centered education since the years of their studies (23). Same goals refer to medical students, who given their appropriate training, their extensive presence in social media world and the rush of their youth are expected to participate in the aforementioned actions since the years of their studies, while forming alongside a promising and conscious next generation of doctors.

However, following numerous studies medical students may have stereotypical opinions regarding psychiatric patients similarly to the general population (24, 25), and often feel awkward when in contact with them (26), believing that they have a poor prognosis (27), and considering that collaborating with them will be extremely stressful (28), emotionally overwhelming (29, 30), and even menacing (31). Even though this negative approach could have been present prior to medical training, it could also have been influenced and shaped through stigmatizing viewpoints expressed by their own instructors (24). Research also reveals that after graduation from medical schools, physicians can exhibit increased stigmatizing perceptions regarding mental illness’ social aspects, such as patients’ both social integration and personal socialization (32).

These medical students’ perceptions are crucial as they directly associate with psychiatric patients’ treatment. More specifically, most health professionals, regardless of specialty, systematically treat patients with co-occurring mental disorders. Graduated medical students who finish their studies without having improved their antecedent perceptions of psychiatry will eventually transform into medical practitioners who feel incompetent or reluctant to address mental illness, therefore sustaining stigmatization, misinformation, and the resulting limited care (33, 34).

Factors such as a higher social life enjoyment among medical students have been linked to increased stigmatizing perceptions (35), while a personal or family history of mental disease seems to incite their compassion toward individuals with a mental health condition (35–37). Surprisingly, it has been further reported that medical students in distress tend to adopt more frequently stigmatizing behaviors toward psychiatric patients than their non-distressed classmates (35). In a survey conducted at the University of Michigan Medical School, students with high scores of self-identified depression repeatedly expressed the opinion that they are viewed as less competent and appeared less likely to seek treatment compared to students with low scores of depression (38, 39).

These findings—except for being the outgrowth of mental distress or disorder—could possibly further reflect the pressure upon medical students by the public’s general perception and high expectation of them supposedly being confident and resilient. This misconception could result in self-stigma that is the incorporation of others’ stereotypes about mental conditions into one’s convictions about oneself (40). Consequently, mental illness and self-stigma, besides affecting the quality of life of medical students, may also result in long-term consequences in regards to the treatment of their future patients (41): as data indicate, medical students with previous psychiatric problems tend to be unwilling to refer patients for treatment if they believe the stigmatization will overshadow the benefits (42).

Finally, a specific question arises: to what extent medical students themselves—the future physicians—are prepared to face the stigma that is hidden behind mental illness and support those suffering from it and calling for respect. Their knowledge, experience, and humanistic opinions about mental health related stigma can serve as veritable tools to fight against this social “disease.” Our study seeks to make an approximate measurement of the presence and degree of this kind of stigmatization among a group of healthcare students, which is expected to play a significant role in the equal perception and treatment of every patient regardless their mental state, as their aspiring physicians (20) whose opinion is publicly perceived as expert (23, 43, 44). It also aims to highlight the areas that call for critical action, both educationally and socially. To achieve these objectives, we employed widely used questionnaires, the analysis of which gave us the chance to detect specific problematic areas and needs for the population studied, as well as to compare them with previous studies on students, healthcare professionals and the general population. This way the researchers of the current study and future researchers have the opportunity to come up with targeted suggestions for further investigation, and educational and social anti-stigma interventions.

## Materials and methods

### Study design

This is a cross-sectional, observational study, aiming to (a) explore the attitudes of undergraduate medical students about mental illness, (b) investigate possible differentiations among them regarding their special characteristics (e.g., demographics, training, and familiarization with mental disorders), and (c) compare them with previous studies on students or on populations with similar characteristics (e.g., similar age, occupational, or educational level). This way, questions will arise about the sufficiency and evaluation of students’ training and the probable impact of labor or contact with patients to one’s attitudes.

In the present study, 324 undergraduate medical students from Aristotle University of Thessaloniki (AUTh), Greece, participated. The School of Medicine of AUTh constitutes the medical school with the highest attendance in the country, counting more than 4.000 registered students in its undergraduate and postgraduate programs (45–47), and bringing together students from all over the country and Cyprus and a minority of foreigners as well, including military medical students.

Notably, the undergraduate curriculum is of 6-year duration, with the initial 2 years principally focused on basic sciences, while contact with clinical experience begins in the spring semester of the third year (sixth semester). During their studies, students receive psychiatric training through a considerable number of elective lessons (available since the first year of education), and—mainly—through mandatory clinical psychiatric practice in their eighth academic semester and optionally in their sixth year of studies as well.

The study was conducted during the spring semester of 2022 from February 1 to May 25 (where third year students had just been introduced into clinical training, fourth year students had just started their psychiatric clinical training and students in their sixth- or higher-year of studies had already completed one or two semesters in clinical Psychiatry), during a difficult time period, where students had to face the personal and training limitations of the COVID-19 pandemic. The relative permission was granted by the headmaster of the School of Medicine of AUTh, after officially informing—via written letter—the general secretary of the school. The questionnaires were distributed mainly through email sent by the general secretary to every single undergraduate student, as well as via a social media platform. Medical students were invited to participate in the research voluntarily and anonymously, having provided informed consent through the initial briefing for the survey on the online platform. The sample of our study was formed from all the answers collected via the electronic questionnaire.

Ethical approval was received from the Scientific Committee of the General Hospital of Thessaloniki “Papageorgiou” Review Board before the collection of data.

### Questionnaires/tools

#### Sociodemographic questionnaire

Participants were invited to provide anonymous demographic information on their gender, family status, and year of medical studies, as well as prior training in clinical psychiatry (Supplementary Table 5).

#### Opinion about Mental Illness scale (OMI)

Respondents were also asked to complete the Opinions about Mental Illness Scale (OMI) (48), originally created by Cohen and Struening in 1959, aiming to assess the viewpoints of healthcare professionals concerning mental illness. The current form of the OMI—which was obtained from profound factor examination of its primary shape of 200 items by more than 8,000 mental health experts—contains 51 statements demonstrated via a six-point Likert-type scale (49). Responses range from 1 (Entirely Agree) to 6 (Entirely Disagree). Factor analysis of the 51 items exposed the following five subscales for the initial English version: A: Authoritarianism, B: Unsophisticated Benevolence, C: Mental Hygiene Ideology, D: Social Restrictiveness, and E: Interpersonal Etiology (48, 49).

The Greek OMI version (Supplementary Table 1), was standardized for the Greek population by Madianos et al. (50), who reported its validity and reliability as well. It follows a modified evaluative scheme (Supplementary Table 2), which stresses the following five factors (20):

- Factor 1: Social Discrimination (SD; 16 items): this factor refers to the identifying features of mental health patients, who are mostly treated as second-class individuals in comparison to those perceived as “normal.” It also contains a hidden belief that psychiatric patients should be treated in an authoritarian manner.
- Factor 2: Social Restriction (SR; 13 items): It portrays the tendency that precautionary actions should be adopted by the society concerning mental health patients. It implicates rejective and coercive convictions referring to penalizations in the course or following a psychiatric hospitalization.
- Factor 3: Social Care (SC; 8 items): This factor contains favorable perspectives about the treatment principles, proposing improvement of quality of care and social assistance.
- Factor 4: Social Integration (SR; 8 items): This one describes the urge to favor equal opportunities in social inclusion of mental health patients in every single facet of public life.
- Factor 5: Etiology (E; 6 items): This factor mentions the opinions about the cause of psychiatric disease, portraying an inclination to assign that to the patients’ relatives.

Statements of OMI are demonstrated in Supplementary Table 1, while those included for the assessment of each of the above factors are presented at Supplementary Table 2 (20).

For each factor, the final score is calculated by adding the scores of all the items contained and subtracting them from a constant number (20, 51). Higher scores demonstrate that the participant inclines more toward the attitude illustrated by every factor (51). In particular, higher scores for factors 1, 2, and 5 represent more stigmatizing and stereotypical beliefs. On the contrary, higher scores for factors 3 and 4 indicate more positive opinions regarding mental disorders and patients suffering from them (20).

The OMI scale has been widely used—both spatially and temporally—among healthcare professionals’ categories, as well as in various populations like undergraduate students, the general population, and psychiatric patients’ relatives (48, 49, 52, 53). Furthermore, the OMI scale has been commonly used in Greece, both for the general population (50, 54), and for subpopulations, including students (51, 55–58) and mental healthcare professionals (20, 25, 59, 60).

#### Social Distance Scale (SDS)

Respondents were invited to complete the Social Distance Scale (SDS) as well (61, 62), a tool often used in stigma research, with good reliability and validity (56, 61–63). It includes seven items (Supplementary Table 3) answered via a four-point Likert-type scale. Example items: “How willing would you feel about working with someone with a mental illness?” “How willing would you feel about renting a room in your home to someone with a mental illness?” The options for the Greek version used range between 0 (Entirely Unwilling) and 3 (Entirely Willing) (63). However, it is noted that the scores were reversed for the statistical analysis process, to be comparable with the results from international literature (20). Total scale scores vary between 0 and 21, by summing the individual scores of all the answers. This scale estimates the social distance the interviewee wishes to keep from a person suffering from a certain condition; in the current study, it calculates the distance that the medical students wish to keep from psychiatric patients (63, 64) with higher scores indicating a stronger will to do so (20).

#### Level of Contact Report (LCR)

The last questionnaire respondents were invited to complete was the Level of Contact Report (LCR-12), a scale initially created by Holmes et al. (64, 65). It is a psychometric self-report test that estimates acquaintance with mental illness. LCR-12 includes 12 statements (Supplementary Table 4) that were derived from other scales employed in stigma research (30) and holds well-reported reliability and validity (65, 66). Each of the statements equates to a particular score (from 1 to 12), depending on the increasing degree of familiarity with mental disorders that it portrays (20, 66). Example items: “I have never observed a person that I was aware had a mental illness.” (rank order score 1), “I have watched a documentary about mental illness.” (score 4), “I suffer from a mental disease” (score 12). Concerning the completion of the scale, participants can select one or more of the 12 declarations, in case they have experienced them before (52, 63). The final score for each respondent is equal to their highest-scoring answer, that is, to the one exhibiting the highest level of familiarity (20, 65, 67).

For all the above questionnaires, the validated Greek version was used (50, 51, 63).

### Statistical analysis

Data were checked for deviations from normality by Kolmogorov–Smirnov test. Comparison of mean scores at OMI subscales (Social Discrimination, Social Restriction, Social Care, Social Integration, and Etiology), SDS, and LCR between categories in sex (male vs. female), year of studies (a. 1, b. 2, c. 3, d. 4, e. 5, f. 6, and g. > 6), family status (h. Married, i. Single, and j. Other), and previous clinical Psychiatry training (k. one semester, l. two semesters, and m. None) were performed with parametric tests in case of normal distribution (*t*-test, ANOVA). Otherwise, non-parametric tests were applied (Mann–Whitney U Test, Kruskal-Wallis test). In case of statistical significance, *post-hoc* analyses were performed, in order for differences in demographics between specific groups to be identified. The same analysis was carried out for some selected items of high interest (items 4, 24, 29, 41, and 51) of the OMI scale. Cronbach’s alpha was also calculated in each subscale of OMI, as well as in SDS scale, in order to assess the influence of each one on the subscales’ internal consistency. Spearman’s correlation was performed in order to assess the relationship between subscale of OMI, SDS, and LCR. An alpha error of 5% (*p* < 0.05) was considered as statistical significance threshold for all analyses. The statistical analyses were performed with SPSS (Version 29, IBM, Armonk, NY, United States).

## Results

### Sample characteristics

In total, 324 subjects were recruited. The subsequent distribution was based on gender: 62% female, 38% male; year of studies: 20.4% 1st, 10.2% 2nd, 13.3% 3rd, 23.3% 4th, 8.3% 5th, 21.0% 6th, and 3.4% >6th; family status: 92.3% single, 1.5% married, and 5.3% other; and previous clinical Psychiatry training in semesters: 38.3% 1 s, 7.4% 2 s, and 54.3% none. Detailed sample characteristics are presented at Supplementary Table 5.

### Cronbach’s alpha

The internal consistency was excellent (>0.7) for Social Discrimination, Social Restriction, and Social Care OMI’s subscales, and SDS and acceptable for Social Integration (0.675) and Etiology (0.654). Deletion of one item did not change the results, with exception of Item 2 (excellent) and Item 3 (unsatisfactory) for Social Integration, as well as Item 1 (excellent) and Item 20 (unsatisfactory) for Etiology. Results are presented at Supplementary Table 8 and Supplementary material 2.

### Spearman correlation

Spearman correlation revealed that Social Discrimination, Social Restriction, and Etiology were positively correlated with SDS. This finding implies that being more willing to interact with people with mental disorders is associated with less discriminative and restrictive attitudes and less stereotypical ideas about the origin of mental illness. Conversely, it indicates that less authoritarian attitudes, and less prejudiced notions regarding the genesis of mental diseases leads to greater readiness to associate with people suffering from them.

Furthermore, Social Discrimination and Social Restriction were negatively correlated with LCR, which means that a higher level of familiarity with mental disorders and patients is linked to a lower presence of discriminative attitudes or approval of restrictive measures, and vice versa. Social Care and Social Integration were positively correlated with LCR. That is, the more one is familiarized with mental disease, the more he endorses the development of an improved social net for psychiatric patients, and the reverse as well. Finally, SDS was negatively correlated with LCR, which indicates that the desire to associate with individuals with mental disorders is directly proportional to the level of intimacy with mental disease and patients.

Respective results are presented at Supplementary Table 8.

### Comparison of OMI subscales

Results are presented at Supplementary Table 6, while the scoring intervals of each subscale are provided in Supplementary Table 11.

#### Social Discrimination (SD)

Analysis for mean scores regarding Social Discrimination revealed statistically significant associations for sex, year of studies and previous Psychiatry training, with males, students in the 1st year and those with no previous Psychiatry training to have the higher (more discriminative) scores. Women presented a quite refined profile compared to men, within the limits of sufficient contradiction to the discriminative notions, while students above the 4th year of studies showed a less authoritarian character more clearly. Notably, all the examined groups in general expressed their strong or only partially doubtful disagreement to the expressed notions that could be considered as a rather satisfactory fact.

#### Social Restriction (SR)

Analysis for mean scores regarding Social Restriction revealed statistically significant associations only for sex, with males having the higher (more restrictive) scores. Nonetheless, it is worth noting that all groups expressed their generous and undoubted disagreement to restrictive measures.

#### Social Care (SC)

Analysis for mean scores regarding Social Care revealed no statistically significant difference in mean scores between groups. This factor was found to be more consistent among the participants, who expressed their explicitly positive attitude about the urgency for better providence for those suffering from mental diseases (mean scores above or below the threshold between “agreement” and “full agreement”).

#### Social Integration (SI)

Analysis for mean scores regarding Social Integration revealed statistically significant associations for year of studies and previous clinical Psychiatry training: sophomores and first year students, and those with no previous clinical Psychiatry experience had the lower scores, indicating the more negative attitude toward patients with mental disorders. All groups demonstrated cautiously supportive beliefs concerning the social inclusion and equal treatment of individuals with mental disorders, while students from the 5th year and above, singles, and those with higher clinical Psychiatry experience appeared slightly more daring in a positive way (mean scores within the spectrum of “agree” with the items included).

#### Etiology (E)

Analysis for mean scores regarding Etiology revealed statistically significant associations for sex, year of studies and previous Psychiatry training, with males, students in the second year and those with no previous Psychiatry training having the higher scores (expressing more stereotypical attitudes). All groups remained rather willing to avoid misconceptions on mental disorders’ etiology (mean scores ranged in the spectrum of “rather disagreement” with the statements under consideration), while students who had completed their 6-year education appeared less prejudiced and only singles stood out more decisively in a more positive way (by entering the spectrum of “disagreement” with the stereotypical beliefs examined).

### Comparison of SDS

Analysis for mean scores regarding SDS revealed statistically significant associations for year of studies, with students in the second year having the higher scores—depicting poorer willingness to associate with people suffering from mental disorders. All groups displayed their probable willingness to interact with psychiatric patients, with sophomores tending to be more ambivalent, in contrast with those with the maximum clinical psychiatric education and even more those who had completed their 6-year educational program, who appeared more decisive to do so. Results are presented at Supplementary Tables 3, 6.

### Comparison of LCR

Analysis for mean scores regarding LCR revealed statistically significant associations for year of studies, with students in the 2nd year having the lower scores (μ:7.70), indicating they are less familiarized with mental illness and patients. Remarkably, all other groups showed a great level of intimacy (rated over 8), which corresponds—at least—to the belief that their job involves providing services and treatment for persons with a mental disease, with questions given a higher rating referring to one’s friends/relatives/family/oneself with a psychiatric history. The higher the year of study and the level of clinical education in Psychiatry, the more intimate (or probably the braver to mention it) the participants appeared. Furthermore, a respective total percentage of 52.6% declared that providing services to psychiatric patients is part of their job, while >41% mentioned a friend or relative with mental health problems and 9% of the participants presented themselves suffering from a mental condition (that constitutes the highest degree of contact report:12). Results are presented at Supplementary Tables 4, 6.

Stereotypical opinions (as measured with OMI) and willingness to interact with people with mental problems (as expressed by SDS) are separately reported for each LCR item at Supplementary Table 7. In terms of Social Care and Etiology, all 12 groups of LCR choices remained quite consistent, while those suffering from a mental problem (item 12) and even more those having a family member with a mental disorder (item 11) displayed more positive and less stereotypical attitudes regarding Social Discrimination, Social Restriction, and Social Integration, and appeared more willing to interact with patients. It was interestingly reported that those who declared to have taken a course on mental illness (item 7), showed the second most favorable opinion about the etiology of mental disease (after the aforementioned group of item 11).

### Comparison of selected items 4, 24, 29, 41, and 51 of OMI scale

The items below were specifically and separately examined (at Supplementary Table 9), due to their distinctness to detect more problematic and stereotypical views (4). They appear to capture major social issues: firstly by broaching essential democratic values and great ethical dilemmas, in which nowadays medical students and future physicians will be called to provide scientific answers (items: 4, 29, and 51); secondly by highlighting the importance of medical confidentiality and the understanding of the dire need to fight social ignorance as medical scientists and mental health experts, in order to dispense people who have suffered from a mental health problem from the burden of hiding it and having to prove themselves and their capacities repeatedly (items: 24, 41).

#### Item 4 (“*Even if psychiatric patients may seem to be okay, they should not be allowed to get married*.”)

It belongs to the items assessing social discrimination. Analysis for mean scores regarding Item 4 revealed statistically significant difference based on previous training on Psychiatry, with the lower scores—which correspond to beliefs more approving of the statement and, as a result, more stigmatizing—to be for those with no training. Nevertheless, all groups expressed a considerable level of disagreement to the statement (μ > 4.76), that is a less discriminative opinion, with singles being slightly more cautious to do so.

#### Item 24 (“*It would be foolish for a woman to marry a man who once had a serious mental illness, even if he appeared to be fully mentally restored*.”)

It is included in items of social discrimination. Analysis for mean scores regarding Item 24 revealed no statistically significant associations. Nonetheless, the individual groups of the participants expressed, in general, quite clearly their disagreement to the above declaration.

#### Item 29 (“*Anyone who is hospitalized in a psychiatric unit should not be allowed to vote*.”)

It constitutes one of the social restriction items. Analysis for mean scores regarding Item 29 revealed statistically significant associations for sex, with the higher scores (which express a greater disagreement to the item) to be for the females. Yet, all the examined groups displayed their disapproval of the above statement, more or less (mean scores within the spectrum of “rather disagree” and “disagree”), while singles appeared a little more reluctant and restrictive compared to others.

#### Item 41 (“*Most women who have been hospitalized in a psychiatric unit should be trusted to look after children*.”)

It is indicative of social integration items. Analysis for mean scores regarding Item 41 revealed no statistically significant difference in mean scores between groups, which ranged in moderate scores (between “rather agree” and “rather disagree”) and preferred safer waters. Students who completed their education (>6th year of studies) seemed slightly more troubled about this item.

#### Item 51 (“*All patients in psychiatric units should be prevented from having children with sterilization*.”)

It is included among the items of social restriction factor. Analysis for mean scores regarding Item 51 revealed no statistically significant difference in mean scores between groups. However, all the individual populations were strongly against the aforementioned notion, expressing their great assurance and respect for patients and democratic principles.

### Presentation of the OMI items with the extreme mean scores and standard deviations

Mean scores and standard deviations for each one of the 51 items of OMI are presented at Supplementary Table 1. The following tables present the items that stood out in the total population by their mean score or their standard deviation (Tables 1, 2).

**Table 1:** Items of minimum and maximum mean scores in OMI analysis.

Items of OMI	Mean score^*^	Std. deviation	ΟΜΙ factor
12. Even though patients in mental hospitals behave in funny ways, it is wrong to laugh at them.	**1.21**	0.68	SC
22. Anyone who tries hard to better himself deserves the respect of others.	**1.45**	0.81	SC
47. Our mental hospitals should be organized in a way to make the patient feel as much as possible as if they are living in their home.	**1.57**	0.89	SC
40. No matter how you look at it, people with serious mental illnesses are no longer real people.	**5.48**	0.87	SR
32. Being hospitalized in a psychiatric clinic is tantamount to failing in real life.	**5.52**	0.9	SR
31. The best way to handle patients in mental hospitals is to keep them behind locked doors.	**5.62**	0.73	SR

^*^Answers rating scale from 1 (Fully Agree) to 6 (Fully Disagree). SD, Social discrimination; SR, Social restriction; SC, Social care; SI, Social integration.

**Table 2:** Items of minimum & maximum SDs in OMI analysis.

Items of OMI	Mean score^*^	Std. deviation	ΟΜΙ factor
12. Even though patients in mental hospitals behave in funny ways, it is wrong to laugh at them.	1.21	**0.68**	SC
31. The best way to handle patients in mental hospitals is to keep them behind locked doors.	5.62	**0.73**	SR
22. Anyone who tries hard to better himself deserves the respect of others.	1.45	**0.81**	SC
19. A heart patient has just one thing wrong with him/her, while a mentally ill person is completely different from other patients.	3.57	**1.39**	SD
48. One of the main causes of mental illness is the lack of moral strength or willpower.	4.01	**1.58**	SD
2. Mental illness is an illness like any other.	3.05	**1.67**	SI

^*^Answers rating scale from 1 (Fully Agree) to 6 (Fully Disagree). SD, Social discrimination; SR, Social restriction; SC, Social care; SI, Social integration.

As shown on the tables, participants expressed positive opinions in a more explicit way regarding people with mental disorders, in matters of social care (with the lowest mean scores that express their agreement with the items) and social restriction (highest mean scores that correspond to one’s disagreement with the statements). They were also found to have given more convergent answers about these factors (as shown by their low standard deviations), but more divergent about some discriminative matters (as expressed by their higher standard deviations).

## Discussion

The present study aimed to evaluate the attitudes on mental illness of medical students at Aristotle University of Thessaloniki, the most populous Medical School of Greece, which students are expected to be the next generation of physicians that will staff the Hellenic National Health System, and other—basically European—health systems as well. Medical students are charged with high expectations for the future of healthcare systems and the establishment of equal provision for all patients, so the evaluation of their current beliefs and the outcomes of their education are of high importance for the following steps.

In Greece, an improvement in the perception of people experiencing or living with a mental disorder has been recorded throughout the last decades among the general public and healthcare population (4), alongside the modernization of the mental healthcare system (68, 69), yet both still lacking. Our study’s goal, apart from presenting medical students’ attitudes toward psychiatric patients, was also to compare them to previous similar studies in student populations (51, 55, 67, 70, 71) and current studies of healthcare personnel (20), and to explore areas for intervention, as well.

In total, our study describes a certain degree of positive attitudes toward people with a mental disorder among medical students of the biggest University of Greece, who interestingly reported a quite high level of contact with mental illness, as well. More specifically, they appeared to almost completely agree with the necessity for measures of high social provision and disagreed with restrictive notions, providing respective answers of high congruency. On the other side, they reported less satisfactorily positive attitudes regarding social discrimination, where their opinions were essentially divergent. Similarly, they expressed themselves less positively concerning the etiology of mental disease, the integration of patients experiencing a mental disorder, and the willingness to interact with them.

In the section below, we summarize the specific features of stigmatization based on participants’ characteristics. Regarding the different groups of medical students, we observe that:

- Sex appeared statistically significant in terms of Social Discrimination, Social Restriction, and Etiology. That indicates that women seemed more sensitive, expressing less stigmatizing notions in the fields more linked to authoritarianism, prejudice, stereotypes, and lack of awareness.
- Year of studies was not considered a statistically significant factor regarding Social Restriction and Social Care, but preclinical students (below the third year) expressed more cautious and less positive opinions concerning Social Discrimination, Social Integration, and Etiology of mental illness. Students of the last years of studies (and students not having yet graduated after the completion of their sixth year education) showed higher desire for interaction with psychiatric patients (as was captured by SDS), while appearing braver and more sensitive in terms of familiarity with mental illness (as shown by LCR).
- Family status did not affect any of the sectors studied in a statistically significant way, yet it should be noted that the vast majority of the participants (>92%) were single.
- Clinical psychiatry training seems to have significantly determined in a more positive and less stigmatizing way the beliefs of the participants, regarding Social Discrimination, Social Integration, and Etiology. It is remarkable that those who declared to have taken a course on mental illness (LCR item 7) appeared to have the second-best opinion regarding the origin of mental illness (after those who stay with a person with a mental disease).

These results should be interpreted cautiously, as prior research has reported the following questionings: the expressed attitudes may differ from the real ones, either due to the factor of social desirability that derives from the implied professional ethos and tends to present less stigmatizing opinions (25), or due to an increased uneasiness for social health and safety (as a result of the professional responsibility “burden”) mixed with insufficient knowledge and familiarity with mental illness that can lead to the choice of expressing more reserved notions (49).

Prior research of Greek and international student populations has reported similar findings to ours: women tend to show a more humanitarian and less stigmatizing profile (37, 70), lower years of studies present more negative attitudes (70), previous contact with psychiatric patients leads to a more friendly and favorable attitude toward them (32, 35, 37, 70, 72) and to a less strong desire for social distance (56). However, few studies of the past described the opposite influence of the factor of sex (57) or of previous personal experience and contact specifically with schizophrenia (25, 67) to one’s opinions and willingness to associate with people living with it.

Comparing our results to previous research of Greek medical students using the same tools and evaluation method, we have reported an increased level of familiarity with mental disease and patients, significantly improved opinions regarding discrimination, less restrictive notions and prejudice regarding the etiology of mental illness, and slightly improved profile regarding social provision and integration (51). These findings are consistent with international literature that describes generally positive beliefs among medical students’ and their amelioration with time (24, 32, 36).

In comparison with a recent study that was conducted in a tertiary University Hospital of Thessaloniki during the same time period (by the same main authors and editor, using the same tools) (20) and had already demonstrated less stigma and prejudices compared to Greek data from previous decades (50, 51, 54, 73–75), we mention the following conclusions, contrasting them with the groups of healthcare professionals with similar characteristics—that is physicians, young people, and those of higher/tertiary education—that showed a more refined, and less stigmatizing profile as well (Supplementary Table 10).

In terms of Social Discrimination and Social Restriction, students showed a significantly better profile than the previous groups, with statistical proximity to the beliefs of the physicians’ group, while they expressed the most positive attitudes among all groups regarding Social Care. Concerning Social Integration and Etiology, students’ attitudes were found within the limits of the scores of the aforementioned groups, with statistical closeness to the beliefs of physicians and higher education graduates for the first factor and to those of physicians and young employees for the latter. Regarding the familiarity with mental illness and people suffering from it, medical students reported a quite high degree of intimacy, yet the level of contact for the specific groups above and the total population of healthcare professionals of the examined study was higher. Nevertheless, medical students appeared significantly more willing to interact with people with mental disease.

The aforementioned conclusions could imply the following points, hypotheses, and suggestions for interventions (educational, occupational, and social), as well as for further research:

More and appropriate educational programs need to take place in healthcare faculties, in order to fight ignorance (as expressed with the scores of Etiology OMI subscale). Education that incorporates useful theoretical knowledge (not a sterile only genetic-based one that is associated with pessimism for one’s prognosis), technological means, and more importantly the experiential learning and interaction with people who can narrate their successful story of recovery from a mental disease (19, 21, 22, 62, 76–79) with emphasis to those healthcare professionals who have experienced a mental disorder (21, 80) is required. As it has been specifically reported by previous research for medical students’ psychiatric education, its frequency and quality characteristics are of high importance (36), as different outcomes have been described for different kinds of psychiatric training (81); education including the beneficial characteristics mentioned above can lead to an improvement of students’ opinions, neutralizing stigma and promoting integration (55, 67, 71, 81), while an obsolete and inappropriate one can result in zero or even negative impact to one’s beliefs (25).

Moreover, light should be shed in additional reasons forming the declined opinions of students (especially in terms of Social Discrimination), as personality, psychopathology, or other characteristics or one’s history could be revealed as significant factors. It would also be of great interest to study separately the opinions of military students (the medical population of whom was included in our study), in order to detect possible differences and reasons behind it.

As for doctors’ more negative opinions compared to medical students’ ones (32), they could have arisen due to their stress and fatigue level, as well as their wider contact with psychiatric patients in their mental or somatic acute phase. The finding could also imply a modification of the previous psychiatric training to a more effective one currently or be indicative of increased understanding and romanticism by younger generations. A possible bias in the comparison between our two studies is the fact that psychiatrists were excluded from the first study—with unclear implications for the results, while potential next-generation psychiatrists were included in the students’ population. In any case, investigating the factors that lead healthcare professionals to adopt more stigmatizing views compared to students, as well as providing opportunities for stress relief, suitable educational methods, and anti-stigma interventions is particularly required.

Considering that the—less positive—attitudes of medical students of the first years may reflect those formed during the secondary education or in public life, further research on minors and general population could bring about useful results. Based on them, an appropriate introduction and familiarization with mental illness in the mandatory education could be adopted, and targeted social campaigns (that make good use of media participation and arts as well) could be effectively organized (82–84).

### Limitations of our study

The study was conducted during the COVID-19 pandemic, the impact of which on students’ health and education needs to be taken into consideration, albeit not being somehow measured; it drastically reduced the clinical education of the students and the interaction with patients, alongside inducing other quarantine restrictions and consequences on their daily life and health (such as sleep and mood disorders or even suicidal ideation). Additional social phenomena that have overwhelmed the Greek current affairs, such as the increasing incidents of violence against women and children during the COVID-19 era, the “Me too” movement, the over decennial financial crisis and the refugee crisis could possibly distress medical students as well and some of them even raise important questions about the mental state of the abovementioned groups or individuals, presumably providing fertile ground for developing mental health stigma notions.

Regarding the sample characteristics, we should mention that the participation rate of military students was not assessed, and their subpopulation—of special characteristics and interest about their views—was not evaluated separately. As for “Family status” factor, we highlight that it was not equally distributed among the available choices (majority of >92% were single).

Concerning the statistical analysis and interpretation, a correction for multiple comparisons was not performed. Lastly, most previous studies were compared descriptively, due to the lack of same tools, evaluation or presentation published.

## Conclusion

It is widely accepted that healthcare professionals and especially doctors are—universally and over time-perceived as the ones who determine the public opinion regarding the formation of mental health-related stigma (23, 43, 44). With an eye to the future generation of physicians, we conducted the current study in medical students of Greece, who expressed clearly approving ideas mainly about social provision and certain disapproval of restrictive measures for psychiatric patients. They appeared rather willing to interact with them, a willingness increasing especially among females, those with clinical experience and psychiatric clinical training. Even though our results indicate an improvement in the perception of people with a mental disorder among the Greek medical students when compared to previous data for students and healthcare professionals, they should not be interpreted in an absolute way, but rather as a tendency. Even though there is hope that the still progressing psychiatric modernization in Greece (68, 69, 81) is followed by a progression in attitudes about mental illness, the slow pace of both (20, 68) could no way bring about complacency, as international literature consistently reports the dangerous—and even fatal—outcomes of poor healthcare access resulting from stigma (22, 25, 85–92). Consequently, the present study is rather a reminder for what needs to be done for current and future doctors in order to “benefit their patients, not to harm or injustice them, and to keep pure and holy both their life and art” as was captured in the Hippocratic Oath thousands of years before (93).

## Data availability statement

The original contributions presented in the study are included in the article/**Supplementary material**, further inquiries can be directed to the corresponding authors.

## Ethics statement

The studies involving humans were approved by Institutional Review Board of the Neurology Clinic, “Papageorgiou” General Hospital of Thessaloniki, Greece (protocol code: 120 /date of approval: 19/02/2021). The studies were conducted in accordance with the local legislation and institutional requirements. The participants provided their written informed consent to participate in this study.

## Author contributions

G-NP, GD, JR, AK, and ID: conceptualization. G-NP, GD, JR, AK, ID, and VS: methodology. VS and ED: software. VS, G-NP, MA, ED, GD, JR, AK, and ID: validation. VS, MA, ED, and JR: formal analysis. G-NP, KA, SS, S-CZ, MA, GD, AK, and ID: investigation. G-NP, KA, MA, SS, S-CZ, and AK: resources. VS, MA, ED, AK, and JR: data curation. G-NP, MA, KA, SS, and S-CZ: writing-original draft preparation. G-NP, MA, KA, AK, and ID: writing-review and editing. G-NP, VS, MA, SS, and S-CZ: visualization. ID, AK, JR, and GD: supervision. AK, ID, G-NP, GD, and JR: project administration. All authors contributed to the article and approved the submitted version.
