# Supplementary material for: Mental illness through the perspective of undergraduate medical students in Greece: a cross-sectional study at Aristotle University of Thessaloniki
Source: Front Psychiatry. 2023 Oct 31;14:1228539. doi: 10.3389/fpsyt.2023.1228539 (PMC10646174; doi:10.3389/fpsyt.2023.1228539)
Supplement: Supplementary file 1 [file Table_1.docx]

**Appendix A**

**Table 1**. Items (51) of the OMI scale and data per item.

| SN | **ITEMS** | **μ** | **SD** |
| --- | --- | --- | --- |
| 1 | Nervous breakdowns usually result when people work too hard. | 3.34 | 1.22 |
| 2 | Mental illness is an illness like any other. | 3.05 | 1.67 |
| 3 | Most individuals in mental hospitals are not dangerous. | 2.86 | 1.19 |
| 4 | Although patients discharged from mental hospitals may seem all right, they should not be allowed to marry. | 4.98 | 0.96 |
| 5 | If parents loved their children more, there would be less mental illness. | 3.7 | 1.39 |
| 6 | It is easy to recognize someone who once had a serious mental illness. | 4.38 | 1.14 |
| 7 | Psychiatric patients let their emotions rule them while normal individuals think about what to do. | 4.32 | 1.3 |
| 8 | People who were once hospitalized in a psychiatric clinic are no more dangerous than the average normal person. | 2.76 | 1.09 |
| 9 | When a person has a problem or worry, it is best not to think about it, but keep busy with more pleasant things. | 4.27 | 1.33 |
| 10 | Although they usually aren't aware of it, many people become mentally ill to avoid the difficult problems of everyday life. | 3.99 | 1.38 |
| 11 | There is something about mental patients that makes it easy to tell them from normal people. | 4.39 | 1.19 |
| 12 | Even though patients in mental hospitals behave in funny ways, it is wrong to laugh at them. | 1.21 | 0.68 |
| 13 | The majority of psychiatric patients are willing to work. | 2.66 | 0.97 |
| 14 | The small children of patients in mental hospitals should not be allowed to visit them. | 4.85 | 1.02 |
| 15 | People who are successful in their work seldom become mentally ill. | 5.37 | 0.85 |
| 16 | People would not become mentally ill if they would avoid bad thoughts. | 4.8 | 1.21 |
| 17 | People in mental hospitals are in many ways like children. | 3.53 | 1.19 |
| 18 | More tax money should be spent in the care and treatment of people with severe mental illness. | 1.94 | 1.01 |
| 19 | A heart patient has just one thing wrong with him, while a mentally ill person is completely different from other patients. | 3.57 | 1.39 |
| 20 | Mental patients come from homes where the parents took little interest in their children. | 4.07 | 1.14 |
| 21 | People with mental illness should never be treated in the same hospital as people with physical illnesses. | 4.34 | 1.31 |
| 22 | Anyone who tries hard to better himself deserves the respect of others. | 1.45 | 0.81 |
| 23 | If our hospitals had very well-trained doctors, nurses and auxiliary staff, many patients would become well enough to live outside of the psychiatric clinics. | 2.65 | 1.13 |
| 24 | A woman would be foolish to marry a man who has had a severe mental illness, even though he seems to have fully recovered. | 4.94 | 1.04 |
| 25 | If the children of mentally ill parents were raised by normal parents, they would probably not become mentally ill. | 4.32 | 1.07 |
| 26 | People who have been patients in a mental hospital will never be their old selves again. | 4.28 | 1.14 |
| 27 | Many psychiatric patients are capable of skilled work, even if they are somehow mentally disturbed. | 2.3 | 0.98 |
| 28 | Our psychiatric clinics are more like prisons than places where the mentally ill can receive necessary care. | 2.43 | 1.15 |
| 29 | Anyone who is in a hospital for a mental illness should not be allowed to vote. | 4.32 | 1.11 |
| 30 | The mental illness of many people is caused by the separation or divorce of their parents during childhood. | 3.92 | 1.18 |
| 31 | The best way to handle patients in mental hospitals is to keep them behind locked doors. | 5.62 | 0.73 |
| 32 | Being hospitalized in a psychiatric clinic is tantamount to failing in real life. | 5.52 | 0.9 |
| 33 | Patients in psychiatric clinics should be given more space for private use. | 2.28 | 0.97 |
| 34 | If a patient in a psychiatric clinic hits someone, he must be punished so that he does not do it again. | 4.32 | 1.14 |
| 35 | If the children of normal parents were raised by mentally ill parents, they would probably become mentally ill. | 4.09 | 1.15 |
| 36 | Every psychiatric clinic should be surrounded by high railings and have security personnel. | 4.83 | 1.21 |
| 37 | The law should allow a woman to divorce her husband as soon as he has been confined to a mental hospital with a severe mental illness. | 3.95 | 1.33 |
| 38 | Psychiatric patients who cannot work because of their mental illness should be given money to live on. | 1.92 | 0.89 |
| 39 | Mental illness is usually caused by some disease of the nervous system. | 2.88 | 1.14 |
| 40 | No matter how you look at it, people with serious mental illnesses are no longer real people. | 5.48 | 0.87 |
| 41 | Most women who were once patients in a mental hospital could be trusted as baby sitters. | 3.62 | 1.11 |
| 42 | Most patients in mental hospitals don't care how they look. | 3.72 | 1.13 |
| 43 | College professors are more likely to become mentally ill than are business men. | 4.48 | 1.09 |
| 44 | Many people who have never been hospitalized in a psychiatric clinic are more sick than many psychiatric patients who have been hospitalized. | 2.71 | 1.21 |
| 45 | Although some psychiatric patients seem okay, it is dangerous to forget even for a moment that they suffer from a mental illness. | 3.35 | 1.31 |
| 46 | Sometimes mental illness is a punishment for bad deeds. | 5.29 | 1.11 |
| 47 | Our mental hospitals should be organized in a way to make the patient feel as much as possible as if he is living in his home. | 1.57 | 0.89 |
| 48 | One of the main causes of mental illness is the lack of moral strength or willpower. | 4.01 | 1.58 |
| 49 | There is little that can be done for patients in a mental hospital except to see that they are comfortable and well fed. | 5.02 | 1.11 |
| 50 | Many mental patients would remain in the hospital until they were well, even if the doors were left unlocked. | 3.4 | 1.22 |
| 51 | All patients in mental hospitals should be prevented from having children by a painless operation (sterilization). | 5.43 | 0.87 |
| SN, serial number of items; μ, Mean scores; SD, Std. deviations. Answers rated from 1 to 6, where 1 stands for Fully agree, 2: Agree, 3: Rather agree, 4: Rather disagree, 5: Disagree, 6: Fully disagree. | | | |

**Table 2.** Calculation method of OMI subscales (according to the Greek Validation by
 Madianos et al., 1987)

| **Factor** | **Analysis Formula** |
| --- | --- |
| F1) Social Discrimination | **82** – (4+6+7+9+11+14+15+16+17+19+21+24+35+42+45+48) |
| F2) Social Restriction | **74** – (26+29+31**+**32+34+36+37+39+40+43+46+49+51) |
| F3) Social Care | **38** – (12+18+22+23+28+33+38+47) |
| F4) Social Integration | **41** – (2+3+8+13+27+41+44+50) |
| F5) Etiology | **32** – (1+5+10+20+25+30) |
| *Numbers given in bold refer to the constant number for each factor (as standardized for the Greek validation),  from which the sum of the scores of the items included is subtracted.  *Numbers inside the parentheses refer to the items of OMI scale, whose scores will be summed. | |

**Table 3.** SDS items and data per item.

| SN | **ITEMS** | **μ** | **SD** |
| --- | --- | --- | --- |
| 1. | How willing would you feel about renting a room in your home to someone with a mental illness? | 1.56 | 0.83 |
| 2. | How willing would you feel about working with someone with a mental illness? | 0.93 | 0.77 |
| 3. | How willing would you feel about having someone with a mental illness as your neighbor? | 0.67 | 0.73 |
| 4. | How willing would you feel about having someone with a mental illness as the caretaker of your children? | 2.23 | 0.77 |
| 5. | How willing would you feel about having your children marry someone with a mental illness? | 1.78 | 0.89 |
| 6. | How willing would you feel about introducing someone with a mental illness to your friends? | 0.71 | 0.73 |
| 7. | How willing would you feel about recommending someone with a mental illness for a job working with someone you know? | 1.09 | 0.81 |
| * SN, serial number of items; μ, Mean scores; SD, Std. deviations * The scores refer to the reversal of the responses given for the Greek version, to be comparable to those of international literature, that is, 0 stands for Absolutely Willing, 1: Rather Willing, 2: Rather Unwilling & 3: Absolutely Unwilling. | | | |

**Table 4.** LCR items and valid percentages (V%)

| **RANK** | **ITEMS** | **V%  per Item** | **LCR Rank V%** |
| --- | --- | --- | --- |
| 1 | I have never observed a person that I was aware had a mental illness. | 17.5 | 0.0 |
| 2 | I have observed a person I believe may have had a mental illness. | 91.9 | 0.3 |
| 3 | I have watched a movie or television show in which a character depicted a person with mental illness. | 97.2 | 4.3 |
| 4 | I have watched a documentary about mental illness. | 61.9 | 2.5 |
| 5 | I have observed persons with a mental illness on a frequent basis. | 39.6 | 1.6 |
| 6 | I have worked with a person who had a mental illness at my place of employment. | 20.2 | 1.2 |
| 7 | I have taken a course during my education related to mental illness. | 70.2 | 13.7 |
| 8 | My job involves providing services/treatment for persons with a mental illness. | 52.6 | 18.9 |
| 9 | A friend of the family has a mental illness. | 41.2 | 13.0 |
| 10 | I have a relative who has a mental illness. | 41.5 | 29.8 |
| 11 | I live with a person who has a mental illness. | 7.7 | 5.6 |
| 12 | I have a mental illness. | 9.0 | 9.0 |
| * Ranking numbers are equivalent to the score of each item.  *** We highlight that the item 7 in the Greek version used (Papakosta-Gaki, 2015) differs from the corresponding   item of the original English version “My job includes providing services to persons with a severe mental illness”.** | | | |

| **Table 5**. Baseline characteristics of the participants | | | | | | | | | |
| --- | --- | --- | --- | --- | --- | --- | --- | --- | --- |
| Year of studies | 1st | 2nd | 3rd | 4^th^ | 5th | 6th | >6th | Total |  |
| n (%) | 66 (20.4) | 33 (10.2) | 43 (13.3) | 76 (23.5) | 27 (8.3) | 68 (21.0) | 11(3.4) | 324 |  |
| Sex, n (%) |  | | | | | | | |  |
| Female | 40 (60.6) | 22 (66.7) | 32 (74.4) | 39 (51.3) | 18 (66.7) | 45 (66.2) | 5 (45.5) | 201 (62) |  |
| Family Status |  | | | | | | | |  |
| Single | 61 (92.4) | 31 (93.9) | 40 (97.6) | 72 (96.0) | 25 (92.6) | 62 (91.2) | 8 (72.7) | 299 (92.3) |  |
| Married | 0 (0) | 0 (0) | 0 (0) | 0 (0) | 0 (0) | 4 (5.9) | 1 (9.1) | 5 (1.5) |  |
| Other | 5 (7.6) | 2 (6.1) | 1 (2.4) | 3 (4.0) | 2 (7.4) | 2 (2.9) | 2 (18.2) | 17 (5.3) |  |
| Clinical Psychiatry training, semesters |  | | | | | | | |  |
| One | 0 (0) | 0 (0) | 2 (4.7) | 60 (78.9) | 25 (92.6) | 33 (48.5) | 4 (36.3) | 124 (38.3) |  |
| Two | 1 (1.5) | 0 (0) | 0 (0) | 0 (0) | 0 (0) | 16 (23.5) | 7 (63.6) | 24 (7.4) |  |
| None | 65 (98.5) | 33 (100) | 41 (95.3) | 16 (21.1) | 2 (7.4) | 19 (27.9) | 0 (0) | 176 (54.3) |  |
| % Valid percentage | | | | | | | | |  |

**Table 6**. Comparison of OMI subscales, SDS and LCR

|  | **OMI** | | | | | **SDS** | **LCR** |
| --- | --- | --- | --- | --- | --- | --- | --- |
|  | Social Discrimination | Social Restriction | Social Care | Social Integration | Etiology |  |  |
|  | Mean (SD) | Mean (SD) | Mean (SD) | Mean (SD) | Mean (SD) | Mean (SD) | Mean (SD) |
| Total Population | 13.54 (11.17) | 13.27 (8.98) | 22.74 (4.56) | 17.79 (5.42) | 8.87 (4.68) | 8.95 (4.23) | 8.71 (2.16) |
| Sex |  |  |  |  |  |  |  |
| Male | 15.80 (11.83) | 14.74(10.38) | 22.08 (5.07) | 17.26 (5.47) | 9.61 (5.08) | 8.84 (4.38) | 8.50 (2.18) |
| Female | 12.16 (10.54) | 12.37 (7.89) | 23.14 (4.18) | 18.11 (5.37) | 8.42 (4.38) | 9.02 (4.14) | 8.84 (2.15) |
| p-value | **0.004^** | **0.006^#^** | 0.061^#^ | 0.123^#^ | **0.032^#^** | 0.694^#^ | 0.189^#^ |
| Year of studies |  |  |  |  |  |  |  |
| a.1 | 17.48 (9.22)^e,f^ | 13.61 (6.78) | 23.42 (3.75) | 16.09(5.20)^e,f,g^ | 10.27 (4.10)^c,d,f,g^ | 9.33 (4.57)^g^ | 8.42 (2.77) |
| b.2 | 16.88 (7.36) | 12.97 (5.63) | 22.94 (3.77) | 16.06 (4.77)^e,f^ | 11.00 (3.49)^c,d,e,f,g^ | 10.55 (3.23)^c,e,f,g^ | 7.70 (2.81)^e,g,f^ |
| c.3 | 11.35 (10.32) | 12.88 (6.62) | 22.05 (4.72) | 17.67 (5.55) ^e,f^ | 8.44 (3.91)^a,b^ | 8.49 (3.55)^b,g^ | 8.70 (2.43) |
| d.4 | 14.46 (8.56) | 13.89 (8.42) | 21.68 (4.27) | 16.66 (4.42) ^e,f^ | 8.26 (3.91)^a,b^ | 9.72 (4.29)^g^ | 8.43 (1.55)^e,f,g^ |
| e.5 | 8.19 (10.02)^a^ | 11.07 (6.52) | 23.56 (4.38) | 20.93(4.89)^a,b,c,d^ | 8.04 (5.27)^b^ | 8.41 (4.19)^b,g^ | 9.37 (1.69)^b,d^ |
| f.6 | 11.62 (14.67)^a^ | 13.74(13.11) | 23.21 (5.31) | 19.99(5.52)^a,b,c,d^ | 8.19 (5.94)^a,b^ | 8.15 (4.10)^b.g^ | 9.37 (1.42)^b,d^ |
| g.>6 | 7.91 (17.32) | 11.91(14.30) | 23.09 (6.96) | 20.18 (7.27)^a^ | 6.18 (5.40)^a,b^ | 4.73 (4.47)^a,b,c,d,e,f^ | 9.82(1.53)^b,d^ |
| p-value | **0.001*** | 0.230^$^ | 0.052^$^ | **<0.001^$^** | **0.001^$^** | **0.002^$^** | **0.007^$^** |
| Family Status |  |  |  |  |  |  |  |
| h.Married | 13.65 (11.14) | 13.29 (8.99) | 22.72 (4.62) | 17.74 (5.35) | 8.92 (4.70) | 8.93 (4.10) | 8.71 (2.17) |
| i.Single | 10.20 (13.04) | 11.60 (8.56) | 25.20 (1.48) | 21.40 (5.41) | 5.80 (2.95) | 7.20 (5.02) | 9.00 (1.00) |
| j.Other | 12.82 (12.56) | 13.88 (8.97) | 23.06 (4.13) | 17.88 (6.47) | 9.35 (5.01) | 9.71 (6.06) | 8.53 (2.50) |
| p-value | 0.764* | 0.714^$^ | 0.322^$^ | 0.287^$^ | 0.232^$^ | 0.541^$^ | 0.986^$^ |
| Semesters with Psychiatry training |  |  |  |  |  |  |  |
| k.1 | 10.94 (10.45)^m^ | 11.86 (6.77) | 22.15 (4.32) | 18.38 (5.19)^m^ | 7.77 (4.75) ^m^ | 9.00 (4.34) | 8.79 (1.59) |
| l.2 | 11.92 (13.80) | 13.42 (9.91) | 23.00 (6.17) | 20.42 (5.75)^m^ | 7.92 (4.61) | 6.83 (4.75) | 9.54 (1.86) |
| m.None | 15.60 (15.60)^k^ | 14.24(10.06) | 23.11 (4.45) | 17.02 (5.39)^k,l^ | 9.77 (4.48) ^k^ | 9.21 (4.02) | 8.54 (2.51) |
| p-value | **0.001*** | 0.084^$^ | 0.06^$^ | **0.005*** | **0.002*** | 0.065^$^ | 0.104^$^ |
| Scoring interval per scale/subscale: Social Discrimination, -14 to 66; Social Restriction, -4 to 61; Social Care, -10 to 30; Social Integration, -7 to 33; Etiology, -4 to 26; SDS, 0 to 21; LCR, 1 to 12.  ^Independent sample t-test  *One-way ANOVA  ^#^ Mann-Whitney-U-Test  ^$^ Kruskal-Wallis-Test  ^a-m^ denotes statistical significance between groups in post-hoc analyses  OMI, Opinions on Mental Illness; SDS, Social Distance Scale; LCR, Level of Contact Report.  Statistically significant values are given in bold. | | | | | | | |

**Table 7.** Demographic characteristics, OMI subscales and SDS data per LCR item.

|  | | |  | **LCR** | | | | | | | | | | | |
| --- | --- | --- | --- | --- | --- | --- | --- | --- | --- | --- | --- | --- | --- | --- | --- |
|  | | |  | Item 1 | Item 2 | Item 3 | Item 4 | Item 5 | Item 6 | Item 7 | Item 8 | Item 9 | Item 10 | Item 11 | Item 12 |
| OMI [μ, (SD)] | | | |  |  |  |  |  |  |  |  |  |  |  |  |
|  | Social Discrimination | | | 14.23 (9.62) | 13.50 (10.94) | 13.24 (10.67) | 12.93 (11.42) | 12.84 (10.78) | 12.72 (11.21) | 11.71 (11.01) | 12.63 (11.95) | 11.88 (10.94) | 11.71 (10.54) | 7.08 (7.46) | 8.34 (9.67) |
|  | Social Restriction | | | 13.52 (6.95) | 13.00 (8.54) | 13.10 (8.42) | 12.64 (9.22) | 12.45 (7.14) | 12.92 (7.88) | 12.51 (8.84) | 12.53 (8.64) | 12.33 (8.93) | 12.47 (8.84) | 10.40 (6.59) | 9.86 (5.32) |
|  | Social Care | | | 22.00 (4.65) | 22.76 (4.59) | 22.65 (4.49) | 22.68 (4.76) | 23.24 (4.30) | 22.68 (4.99) | 22.63 (4.56) | 23.18 (4.36) | 23.04 (4.34) | 23.23 (4.34) | 23.84 (3.78) | 23.24 (3.69) |
|  | Social Integration | | | 17.02 (4.43) | 17.84 (5.48) | 17.81 (5.35) | 18.37 (5.31) | 18.17 (5.60) | 19.17 (5.22) | 18.53 (5.30) | 18.31 (5.59) | 18.63 (5.41) | 18.57 (5.56) | 20.28 (5.25) | 20.14 (5.21) |
|  | Etiology | | | 8.98 (4.15) | 8.78 (4.62) | 8.82 (4.54) | 8.85 (4.72) | 8.79 (4.60) | 9.45 (4.91) | 8.40 (4.76) | 8.60 (4.69) | 8.80 (4.69) | 8.84 (4.86) | 8.16 (4.50) | 9.14 (4.74) |
| SDS [μ, (SD)] | |  | |  |  |  |  |  |  |  |  |  |  |  |  |
|  | | | | 8.64 (3.98) | 9.02 (4.28) | 8.95 (4.23) | 8.65 (4.18) | 8.50 (4.58) | 8.18 (4.09) | 8.64 (4.13) | 8.75 (4.45) | 8.16 (4.22) | 8.05 (4.17) | 5.76 (4.04) | 7.62 (4.26) |
| μ: Mean Scores; SD: Std. Deviations | | | | | | | | | | | | | | | |

| **Table 8**. Spearman's correlation for association between OMI Subscales, SDS and LCR. | | | | |
| --- | --- | --- | --- | --- |
| OMI/Subscales | SDS | | LCR | |
|  | r_s_ | p-value | r_s_ | p-value |
| Social Discrimination | 0.546 | **<0.001** | -0.204 | **<0.001** |
| Social Restriction | 0.434 | **<0.001** | -0.181 | **0.001** |
| Social Care | -0.229 | **<0.001** | 0.160 | **0.004** |
| Social Integration | -0.556 | **<0.001** | 0.201 | **<0.001** |
| Etiology | 0.272 | **<0.001** | 0.015 | 0.794 |
| SDS | ΝΑ | ΝΑ |  |  |
| LCR | -0.216 | **<0.001** |  |  |
| OMI, Opinions on Mental Illness; SDS, Social Distance Scale; LCR, Level of Contact Report;  r_s_, Spearman's correlation coefficient; NA, non-applicable  Statistically significant values are given in bold | | | | |

**Table 9**. Comparison of items 4, 24, 29, 41, and 51 of OMI scale

|  | **OMI** | | | | |
| --- | --- | --- | --- | --- | --- |
|  | Item 4 | Item 24 | Item 29 | Item 41 | Item 51 |
| Sex | Mean (SD) | Mean (SD) | Mean (SD) | Mean (SD) | Mean (SD) |
| Male | 4.89 (0.90) | 4.93 (0.94) | 4.12 (1.18) | 3.66 (1.13) | 5.44 (0.87) |
| Female | 5.04 (0.98) | 4.95 (1.09) | 4.44 (1.04) | 3.59 (1.08) | 5.45 (0.87) |
| p-value | 0.052**^#^** | 0.438^#^ | **0.018^#^** | 0.523^#^ | 0.959^#^ |
| Year of studies |  |  |  |  |  |
| a.1 | 4.76 (0.99) | 4.82 (1.14) | 4.27 (1.24) | 3.77 (1.20) | 5.36 (0.88) |
| b.2 | 4.88 (0.89) | 5.00 (0.70) | 4.27 (1.12) | 3.61(0.96) | 5.27 (1.00) |
| c.3 | 5.00 (1.02) | 5.02 (0.91) | 4.48 (0.91) | 3.60 (1.01) | 5.47 (0.79) |
| d.4 | 4.96 (0.85) | 4.81 (1.01) | 4.03 (1.07) | 3.81 (1.04) | 5.43 (0.82) |
| e.5 | 5.27 (0.87) | 4.89 (1.01) | 4.37 (1.24) | 3.30 (1.03) | 5.52 (0.64) |
| f.6 | 5.12 (0.92) | 5.11 (1.14) | 4.53 (1.05) | 3.53(1.15) | 5.53 (0.86) |
| g.>6 | 5.09 (1.07) | 5.18 (1.16) | 4.73 (1.42) | 2.82 (1.16) | 5.36 (1.50) |
| p-value | 0.067^$^ | 0.259^$^ | 0.076^$^ | 0.100^$^ | 0.774^$^ |
| Family Status |  |  |  |  |  |
| h.Married | 5.00 (0.93) | 4.95 (1.01) | 4.33 (1.08) | 3.63 (1.10) | 5.44 (0.84) |
| i.Single | 4.20 (1.30) | 5.20 (0.83) | 3.80 (1.30) | 3.00 (1.00) | 5.80 (0.44) |
| j.Other | 5.00(1.32) | 4.76 (1.52) | 4.18 (1.55) | 3.71 (1.26) | 5.12 (1.36) |
| p-value | 0.227^$^ | 0.895**^$^** | 0.672^$^ | 0.437^$^ | 0.497^$^ |
| Semesters with Psychiatry training |  |  |  |  |  |
| k.1 | 5.15 (0.84)^m^ | 4.94 (1.05) | 4.33 (1.12) | 3.59 (1.15) | 5.52 (0.80) |
| l.2 | 5.04(1.16) | 5.25(1.03) | 4.42(1.34) | 3.58(1.28) | 5.29 (1.19) |
| m.None | 4.85 (0.99)^k^ | 4.90 (1.01) | 4.30 (1.06) | 3.65 (1.04) | 5.39 (0.87) |
| p-value | **0.02^$^** | 0.163^$^ | 0.907^$^ | 0.976^$^ | 0.291^$^ |
| ^#^ Mann-Whitney-U-Test  ^$^ Kruskal-Wallis-Test  ^a-m^ denotes statistical significance between groups in post-hoc analyses  Statistically significant values are given in bold | | | | | |

**Table 10.** Comparison of students’ population with subgroups of healthcare professionals with similar characteristics (doctors, young employees, graduates of higher education).

|  | **OMI** | | | | | **SDS** | **LCR** |
| --- | --- | --- | --- | --- | --- | --- | --- |
|  | Social Discrimination | Social Restriction | Social Care | Social Integration | Etiology |  |  |
|  | Mean (SD) | Mean (SD) | Mean (SD) | Mean (SD) | Mean (SD) | Mean (SD) | Mean (SD) |
| Medical Students total population | 13.54 (11.17) | 13.27 (8.98) | 22.74 (4.56) | 17.79 (5.42) | 8.87 (4.68) | 8.95 (4.23) | 8.71 (2.16) |
| Physicians | 14.49 (9.70) | 13.56 (7.86) | 20.06 (4.03) | 18.02 (4.17) | 8.71 (4.39) | 10.60 (4.23) | 9.31 (1.34) |
| Young healthcare professionals (<30y) | 18.93 (10.41) | 16.67 (8.56) | 20.34 (3.87) | 16.45 (4.67) | 8.68 (4.99) | 10.63 (4.41) | 8.97 (1.66) |
| Higher Education Graduates | 17.40 (11.94) | 15.02 (9.81) | 20.38 (4.25) | 18.01 (3.99) | 9.16 (5.19) | 11.07 (4.44) | 9.02 (1.58) |
| Healthcare professionals total population | 22.99 (12.08) | 17.45 (9.07) | 21.04 (4.12) | 16.38 (4.68) | 9.80 (4.95) | 11.68 (4.28) | 8.82 (1.73) |
| Values refer to the findings of the survey presented at the original article “**Mental health-related stigma discrimination and prejudices among Greek healthcare professionals**”, which was conducted in a healthcare establishment in the same place (Thessaloniki) and time (2021-22), by the same main authors (*doi: 10.3389/fpsyt.2022.1027304*).  Scoring interval per scale/subscale: Social Discrimination, -14 to 66; Social Restriction, -4 to 61; Social Care, -10 to 30; Social Integration, -7 to 33; Etiology, -4 to 26; SDS, 0 to 21; LCR, 1 to 12. | | | | | | | |

**Table 11.** Scoring intervals of OMI subscales

| **OMI FACTORS** | **SCORING INTERVALS** | | | | | |
| --- | --- | --- | --- | --- | --- | --- |
|  | Clearer disagreement | | Ambivalence | | Clearer Agreement | |
|  | Fully disagree | Disagree | Rather Disagree | Rather Agree | Agree | Fully Agree |
| Soc. Discrimination | -14 -0.67 12.67 26 39.33 52.67 66 | | | | | |
| Social Restriction | -4 6.83 17.76 28.5 39.33 50.17 61 | | | | | |
| Social Care | -10 -3.33 3.33 10 16.67 23.33 30 | | | | | |
| Social Integration | -7 0.33 6.33 13 19.66 26.33 33 | | | | | |
| Etiology | -4 1 6 11 16 21 6 | | | | | |
