# Supplementary material for: Mental illness through the perspective of undergraduate medical students in Greece: a cross-sectional study at Aristotle University of Thessaloniki
Source: Front Psychiatry. 2023 Oct 31;14:1228539. doi: 10.3389/fpsyt.2023.1228539 (PMC10646174; doi:10.3389/fpsyt.2023.1228539)
Supplement: Supplementary file 2 [file Table_2.docx]

**Deletion of one item analysis:**

| **OMI SOCIAL DISCRIMINATION:** | |
| --- | --- |
| Total | 0.843 |
| **Item** | **Cronbach's Alpha if Item Deleted** |
| 4 | 0.829 |
| 6 | 0.836 |
| 7 | 0.833 |
| 9 | 0.836 |
| 11 | 0.829 |
| 14 | 0.836 |
| 15 | 0.833 |
| 16 | 0.831 |
| 17 | 0.84 |
| 19 | 0.841 |
| 21 | 0.831 |
| 24 | 0.838 |
| 35 | 0.835 |
| 42 | 0.838 |
| 45 | 0.831 |
| 48 | 0.834 |

| **OMI SOCIAL RESTRICTION:** | |
| --- | --- |
| Total | 0.745 |
| **Item** | **Cronbach's Alpha if Item Deleted** |
| 26 | 0.723 |
| 29 | 0.725 |
| 31 | 0.714 |
| 32 | 0.704 |
| 34 | 0.742 |
| 36 | 0.723 |
| 37 | 0.731 |
| 39 | 0.782 |
| 40 | 0.708 |
| 43 | 0.75 |
| 46 | 0.73 |
| 49 | 0.726 |
| 51 | 0.706 |

| **OMI SOCIAL CARE:** | |
| --- | --- |
| Total | 0.704 |
| **Item** | **Cronbach's Alpha if Item Deleted** |
| 12 | 0.684 |
| 18 | 0.668 |
| 22 | 0.67 |
| 23 | 0.694 |
| 28 | 0.696 |
| 33 | 0.665 |
| 38 | 0.663 |
| 47 | 0.666 |

| **OMI SOCIAL INTEGRATION:** | |
| --- | --- |
| Total | 0.675 |
| **Item** | **Cronbach's Alpha if Item Deleted** |
| 2 | 0.711 |
| 3 | 0.594 |
| 8 | 0.618 |
| 13 | 0.654 |
| 27 | 0.622 |
| 41 | 0.625 |
| 44 | 0.671 |
| 50 | 0.661 |

| **OMI ETIOLOGY:** | |
| --- | --- |
| Total | 0.654 |
| **Item** | **Cronbach's Alpha if Item Deleted** |
| 1 | 0.706 |
| 5 | 0.603 |
| 10 | 0.613 |
| 20 | 0.55 |
| 25 | 0.607 |
| 30 | 0.571 |

| **SDS:** | |
| --- | --- |
| Total | 0.879 |
| **Item** | **Cronbach's Alpha if Item Deleted** |
| 1 | 0.868 |
| 2 | 0.856 |
| 3 | 0.865 |
| 4 | 0.862 |
| 5 | 0.856 |
| 6 | 0.863 |
| 7 | 0.86 |

| **LCR:** | |
| --- | --- |
| Total | 0.525 |
| **Item** | **Cronbach's Alpha if Item Deleted** |
| LCR1 | 0.618 |
| LCR2 | 0.508 |
| LCR3 | 0.519 |
| LCR4 | 0.52 |
| LCR5 | 0.465 |
| LCR6 | 0.485 |
| LCR7 | 0.484 |
| LCR8 | 0.499 |
| LCR9 | 0.437 |
| LCR10 | 0.455 |
| LCR11 | 0.486 |
| LCR12 | 0.515 |
